# Development and evaluation of automated ultrasonographic detection of bladder diameter for estimation of bladder urine volume

**DOI:** 10.1371/journal.pone.0219916

**Published:** 2019-09-05

**Authors:** Masaru Matsumoto, Takuya Tsutaoka, Koichi Yabunaka, Mayumi Handa, Mikako Yoshida, Gojiro Nakagami, Hiromi Sanada

**Affiliations:** 1 Department of Imaging Nursing Science, Graduate School of Medicine, The University of Tokyo, Bunkyo-ku, Tokyo, Japan; 2 Imaging Technology Center, Research & Development Management Headquarters, FUJIFILM Corporation, Minato-ku, Tokyo, Japan; 3 Global Nursing Research Center, Graduate School of Medicine, The University of Tokyo, Bunkyo-ku, Tokyo, Japan; 4 Marketing Planning Group, Ultrasound Promotion Department, FUJIFILM Medical Corporation, Minato-ku, Tokyo, Japan; 5 Department of Gerontological Nursing/Wound Care Management, Graduate School of Medicine, The University of Tokyo, Bunkyo-ku, Tokyo, Japan; International University of Health and Welfare, School of Medicine, JAPAN

## Abstract

Bladder urine volume has been estimated using an ellipsoid method based on triaxial measurements of the bladder extrapolated from two-dimensional ultrasound images. This study aimed to automate this process and to determine the accuracy of the automated estimation method for normal and small amounts of urine. A training set of 81 pairs of transverse and longitudinal ultrasound images were collected from healthy volunteers on a tablet-type ultrasound device, and an automatic detection tool was developed using them. The tool was evaluated using paired transverse/longitudinal ultrasound images from 27 other healthy volunteers. After imaging, the participants voided and their urine volume was measured. For determining accuracy, regression coefficients were calculated between estimated bladder volume and urine volume. Further, sensitivity and specificity for 50 and 100 ml bladder volume thresholds were evaluated. Data from 50 procedures were included. The regression coefficient was very similar between the automatic estimation (β = 0.99, R^2^ = 0.96) and manual estimation (β = 1.05, R^2^ = 0.97) methods. The sensitivity and specificity of the automatic estimation method were 88.5% and 100.0%, respectively, for 100 ml and were 94.1% and 100.0%, respectively, for 50 ml. The newly-developed automated tool accurately and reliably estimated bladder volume at two different volume thresholds of approximately 50 ml and 100 ml.

## Introduction

The term lower urinary tract symptoms (LUTS), which includes voiding and storage symptoms, has been universally recognized since the standardization of the urinary tract function terminology in 2002 [1]. The current guidelines specifically recommend the clinical use of residual urine volume to determine the type of LUTS, with cut-off residual urine volumes of 50 or 100 ml in the elderly and adults, respectively, for determining the presence or absence of urinary tract obstruction [2,3]. Therefore, accurate estimation in clinical settings of bladder urine volume near or below 100 ml without underestimation is imperative for determining the type of LUTS.

Using ultrasonography (US), bladder urine volume can be effectively and accurately estimated. In Japan, the estimation of bladder urine volume using US has been an essential skill for nurses at bedside because continence care including using US assessment has been covered by national health insurance. Currently, there are several portable US devices available for estimation of bladder urine volume worldwide [4–7]; however, US devices that estimate bladder volume without direct visualization of the bladder fail to accurately distinguish among structures such as pelvic cysts and the bladder [8]. US devices that estimate bladder volume via direct visualization are less variable than their counterparts that do not use direct visualization [9]. Thus, US with real-time direct visualization of the bladder is the best option for accurate estimation of bladder urine volume.

There are two main methods of measuring bladder urine volume from two-dimensional (2D) US images: prolate ellipsoid method and double area method [10,11]. Although there are devices that can automatically measure bladder urine volume using the double area method [9,12], they are exclusive devices with limited applications. The ellipsoid method first determines the transverse, anteroposterior, and superoinferior diameter lengths of the bladder from 2D US images and then calculates volume based on a mathematical formula [10,11]. This method can be used in many current US devices and can accurately measure a smaller bladder urine volume than the double area method [13], although most need an operator to manually select and measure the three diameters of the bladder, thus warranting training time for operators to acquire the skill. Similarly, manual operation requires extended time to display measured values and have low usability.

The aim of this study was (1) to develop a new tool for conventional 2D US imaging with a function that automatically determines the three diameters of the bladder from the images and accurately calculates bladder urine volume and (2) to validate the accuracy of the developed tool when bladder urine volume is low, i.e., below 100 ml. In this study, we evaluated the validity of the newly-developed tool in measuring voided urine volume of healthy adults without residual urine as the true value.

## Material and methods

### Development of an automatic detection tool

**Fig 1:**
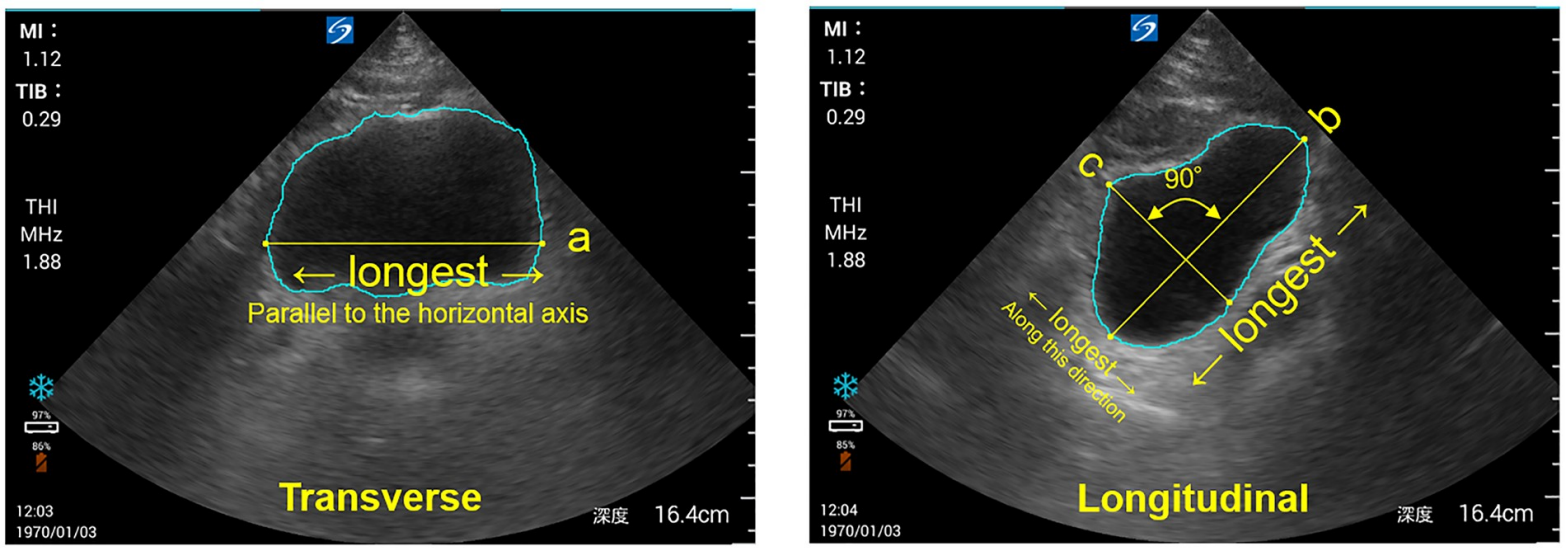
Measurements of three axial diameters on two-dimensional images. a (Transverse section): Two points on the bladder contour with the longest distance on a line parallel to (or small slope against) the horizontal axis. b (Longitudinal section): Two points on the bladder contour with the longest distance. c: Two points on the bladder contour with the longest distance on a line orthogonal to line b.

We developed an automatic bladder volume estimation tool comprising a bladder area extractor and diameter measurer using an artificial intelligence approach. The bladder area extractor was built using fully convolutional networks (FCNs) [14], a recognized deep learning technique for semantic segmentation. We trained the bladder area extractor with a training set of images as described below (see *Training and validation dataset*). The diameter measurer was simply implemented to ensure that the diameters of each cross-section were maximized (Fig 1).

**Fig 2:**
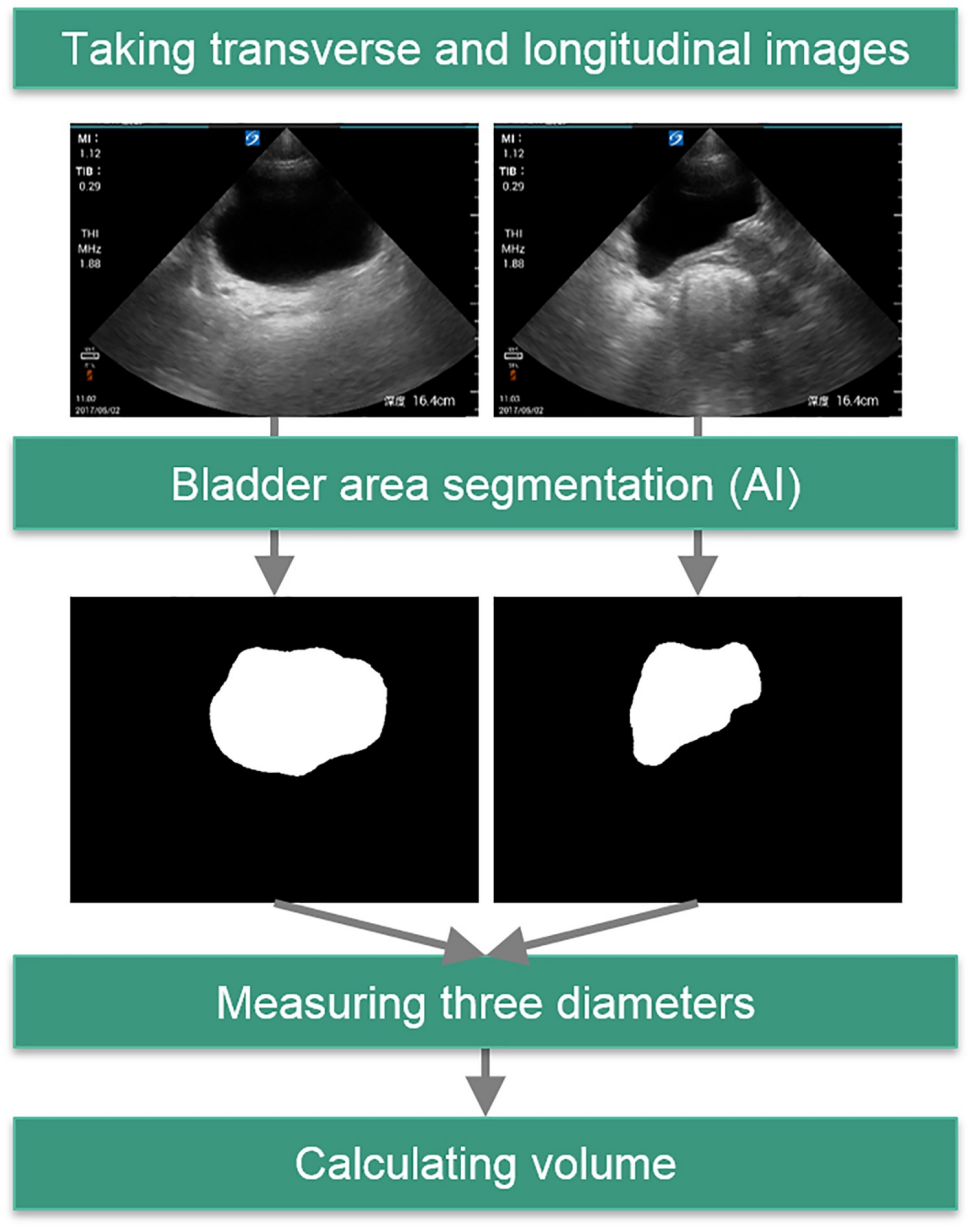
Methodological overview: Automated bladder volume measurement. Users collect one transverse and longitudinal image each, and the subsequent processing is automated. Bladder area segmentation is performed by a deep learning algorithm, measurement of the three diameters is performed by ellipse fitting, and bladder volume is mathematically calculated as an ellipsoid. Even if the bladder protrudes from the view, the correct value can be determined by an artificial intelligence (AI) approach by training under expert advice.

The methodological overview delineating the measurement of automated bladder volume is depicted in Fig 2.

### FCN deep learning

Deep learning, a powerful machine learning technique, can approximate complicated mapping of an input/output space without special preprocessing. The convolutional neural network (CNN) is a deep learning model commonly used in the field of image recognition because it delivers the best performance in various tasks related to object detection and semantic segmentation [15,16]. FCN is a typical semantic segmentation method based on CNNs and comprises an encoder that extracts the image feature and a decoder that estimates the label map from the extracted feature. For bladder area extraction, we used the same FCN model for transverse and longitudinal sections. Considering the issue of data shortage, we used a VGG16-like CNN pre-trained by general images as the encoder. The decoder was composed of FCN-8s as described previously [17]. The number of channels in each convolutional layer of VGG16-like CNN was one-quarter of a VGG16.

### Implementation

Input images were resized to 512 × 384; brightness was changed; and contrast change and horizontal flip were randomly applied as data augmentation. We used stochastic gradient descent with momentum for 200 epochs and adjusted hyperparameters by random search. Learning rates were initialized to 1e-4 and divided by 10 for each of 50 epochs. Our source codes were written based on Keras, and our experiments ran on a single NVIDIA GTX 1080Ti.

### Training and validation dataset

As the training set, 81 pairs of transverse and longitudinal US images were collected using the Sonosite iViz ultrasound device (FUJIFILM SonoSite, WA, USA) from healthy volunteers by a sonographer with over 10-year clinical experience. Intraclass correlation coefficients of estimated volume using images obtained with this device was 0.99.

After training, data from 23 procedures involving a total of 18 healthy volunteers (age: 23–62 years) with voided volume of <150 ml were collected for the validation set. The US operator and device were the same as those used for the training set data. The protocol used was as follows: the procedures were performed 30–60 min following the final urination and transverse and longitudinal US images of the participants’ bladders were obtained using the US device less than 10 min prior to the next urination. The voided urine volume was measured by another blinded researcher using a graduated cylinder (1-mm scale) and reported as the voided volume (ml).

### Evaluation of the automatic detection tool

#### Subjects

Healthy adult volunteers aged between 20 and 64 years were recruited as the study participants. Exclusion criteria included the presence of at least one voiding symptom and history of urological disease. Written informed consent was obtained from all participants prior to their enrollment according to the tenets of the World Medical Association Declaration of Helsinki. This study was approved by the Ethical Committee of the Graduate School of Medicine, The University of Tokyo, Japan (No. 11525). We have received consent to publish US images.

#### Procedure

Transverse and longitudinal US images were collected less than 3 min prior to urination. The participants then voided, following which their urine was collected. Next, the presence or absence of residual urine was confirmed using the ultrasonography. This procedure was performed twice at 30-min intervals for each participant unless consent was not provided for a second procedure, in which case only the first procedure was performed.

In total, data were collected from 54 procedures involving 28 participants. Data from four procedures were excluded from the analysis because residual urine volume was detected; hence, the data from 50 procedures involving a total of 27 participants (14 men, 25–56 years of age; 13 women, 22–54 years of age) were finally included in the analysis.

#### Analysis

For confirming accuracy, regression coefficients (without intercept) and R^2^ values were calculated between the calculated bladder volume and actual urine volume (manually measured using a measuring cylinder). Sensitivity and specificity in discriminating (thresholds of 50 and 100 ml) were also calculated to validate the accuracy of the tool in detecting voiding dysfunction based on urine volume. To compare manual measurement by a sonographer (considered the gold standard of clinical non-invasive bladder urine volume measurement) with the newly-developed tool, bladder urine volume for three bladder diameters was calculated using the automated detection method and the manual detection method by sonographer.

## Results

**Fig 3:**
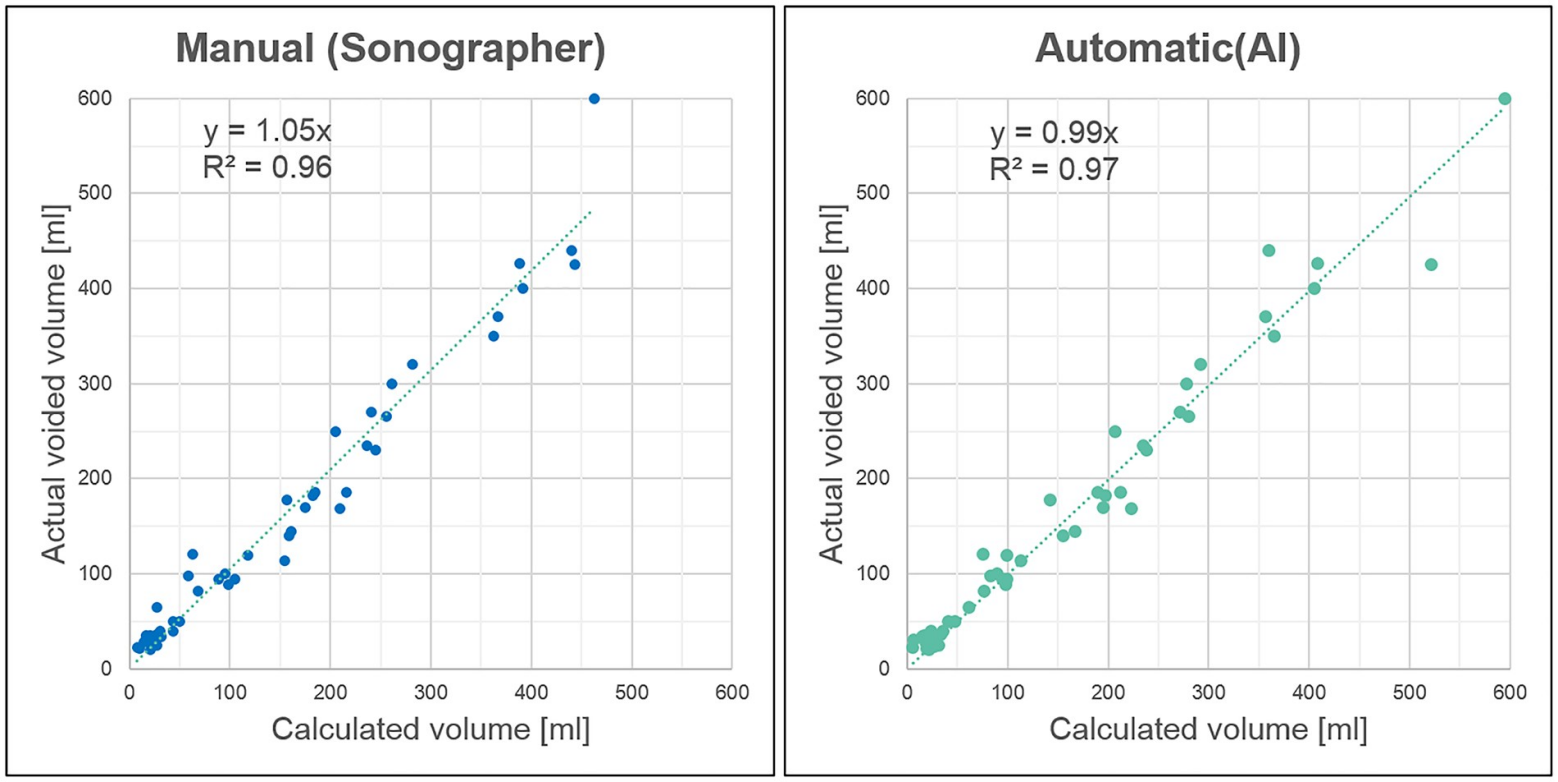
Scatter plots of the actual voided volume versus sonographer-calculated (manual) and AI-calculated (automatic) volumes. Dotted horizontal lines indicate the approximate straight line of the regression equation shown in the square.

Scatter plots of actual voided volume versus manually calculated volume (sonographer-calculated) or automatically calculated volume are shown Fig 3. The regression coefficient was closest to 1 (1 representing perfect accuracy) with the automatic estimation method (β = 0.99, R^2^ = 0.96) and that with the manual estimation method was also close to 1 (β = 1.05, R^2^ = 0.97).

The sensitivity and specificity for the automatic estimation method of actual voided volume at a threshold of 100 ml were 88.5% and 100.0%, respectively and those of the manual estimation method were 92.3% and 95.8%, respectively (Table 1).

**Table 1:** Confusion matrix of actual voided volume versus manually calculated and automatically calculated volumes (threshold: 100 ml).

		Actual voided volume	
		≥100 ml	<100 ml
**Manual**	≥100 ml	24	1
	<100 ml	2	23
**Automatic** **(AI)**	≥100 ml	23	0
	<100 ml	3	24

The sensitivity and specificity of the automatic estimation method at the 50 ml threshold were 94.1% and 100.0%, respectively, and those of manual estimation method were 94.1% and 100.0%, respectively (Table 2).

**Table 2:** Confusion matrix of actual voided volume versus manually calculated and automatically calculated volumes (threshold: 50 ml).

		Actual voided volume	
		≥50 ml	<50 ml
**Manual**	≥50 ml	32	0
	<50 ml	2	16
**Automatic** **(AI)**	≥50 ml	32	0
	<50 ml	2	16

## Discussion

In this study, we developed a new tool to automatically estimate the three axial diameters of the bladder from 2D US images, which was found to be highly accurate in estimating the actual voided urine volumes of approximately 50 ml and 100 ml. Although many reports validate the accuracy of US-based bladder urine volume measurement [4–7,9–12], few reports focus on small volumes [18].

**Fig 4:**
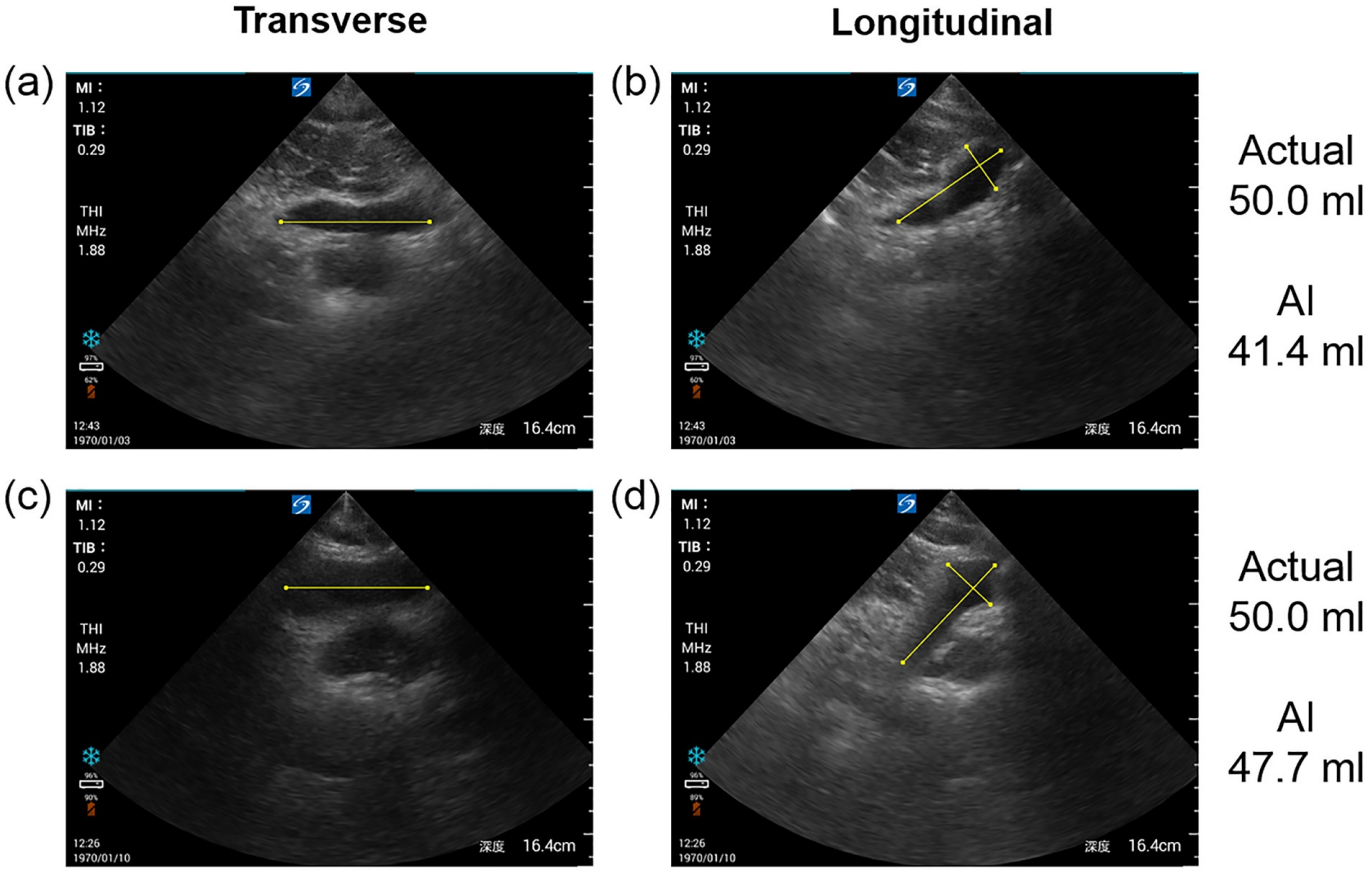
Two cases of false negatives. (a, b) Transverse and longitudinal ultrasound (US) images of the same male aged approximately 20 years. (c, d) transverse and longitudinal US images of the same male aged approximately 30 years. Actual: Urine volume actually urinated by the subject. AI: Value of bladder urine volume estimated by automatic estimation tool. Yellow lines are detected by AI as bladder diameters.

Diagnosing voiding dysfunction is substantially assisted by precise measurements of bladder urine volume by US. In a previous study using 2D US, the sensitivity of estimated urine volume at a 60 ml threshold was 100.0% but that at a 110 ml threshold was 57.9% [18]. Compared with previous studies [18] the sensitivity of our automated tool appears to be good and is superior at the 100 ml threshold. There were two false negatives at 50 ml, but as shown in Fig 4, the caliper almost precisely identified the diameter of the bladder. Because bladder urine volume was less than the 60 ml threshold examined in the previous study, we believe that there was error from the true value at 50 ml. However, because the sensitivity and specificity of this automatic estimation method were equivalent to the expert sonographer’s result, this new tool appears equally viable for estimating bladder urine volume and judging the presence of voiding dysfunction. We expect that nurses can use this new tool that can be easily and accurately measured for point-of-care by not expert in the future.

The ellipsoid method in the current use has two primary issues: accuracy of the estimate depends on the manual technique used to detect the diameter of the bladder and high demands on time and repeated procedures. Automation can solve the latter problem; however, we used US images collected by experts for machine learning and evaluation data. Therefore, presently, our tool is not a tool that can easily and accurately be used to measure urine volume in the bladder. Accurate measurement of small amount of bladder urine volume is necessary to accurately determine the type of LUTS. This study contributes to accurate measurement of bladder urine volume using US and to be able to improve the speed with which clinicians can determine the type of LUTS. From the high sensitivity demonstrated by this study, it will not be underestimated the actual residual urine. In the future, further study that evaluate of validation of our new tool in general medical staff other than sonographer is needed. Furthermore, it is always necessary to standardize the scanning parameters and techniques to allow broader use of the automatic detection support function.

This study has three limitations. First, all US images were acquired by one sonographer, and it remains unclear whether those with comparatively less qualification and/or experience with ultrasound can obtain similar results. In addition, for the current system, the development of educational programs for nurses or other medical professionals will be needed. Second, the subjects were limited to healthy adults, and the efficacy in patients with LUTS remains unclear. However, we thought it was appropriate that this study was conducted with healthy adults. Patients with LUTS may have residual urine due to the influence of aging and the effects of anticholinergics used for treatment. In subjects with residual urine, it is difficult to validate the voiding volume as a true value. The drainage of bladder urine by a catheter for research is invasive and should be avoided. Therefore, in this study, we chose the void volume of healthy adults without residual urine as a true value. In the future, when comparing manual and automated methods to detect bladder diameters, patients with LUTS should be included.

Third, the new tool is not a built-in device in the US, thus requiring a PC to be connected. Although processing time on a PC is only approximately 1 s, the time needed to extract data from the US and transfer it to the PC slows the system down. In the near future, it is hoped that development of a hand-held US device incorporating the automatic estimation tool will occur. Overcoming these limitations will facilitate many health professionals to easily estimate bladder urine volume quickly and accurately.

In conclusion, we successfully developed an automated estimation tool that could determine all three axial bladder diameters and calculate bladder urine volume of approximately 50 ml and 100 ml as accurately as a manual system with high specificity and sensitivity. This system is comparable to expert manual detection methods and may improve the speed with which clinicians can determine the type of LUTS.
